# Examining Penetration and Residual Depth in Modern Acrylic Foldable Intraocular Lenses: A Laboratory Study Using Differential Interference Contrast Microscopy to Compare Hydrophilic and Hydrophobic Materials

**DOI:** 10.7759/cureus.70383

**Published:** 2024-09-28

**Authors:** Andreas F Borkenstein, Eva-Maria Borkenstein

**Affiliations:** 1 Ophthalmology, Borkenstein and Borkenstein, Private Practice at Privatklinik Kreuzschwestern, Graz, AUT

**Keywords:** residual depth, penetration depth, nanoindentation, damages in intraocular lenses, scratch test

## Abstract

Introduction

The material of modern intraocular lenses must meet the highest standards and fulfill various requirements. It is crucial that the material shows the best biocompatibility and should be flexible for an uncomplicated implantation process through small corneal incisions but also sufficiently rigid for good stability and centering in the capsular bag. In addition, the optic must remain clear for life and retain the best optical properties.

Methods

In this laboratory experiment, we performed scratch tests for the mechanical assessment of acrylic intraocular lenses. The aim was to determine differences in the behavior in regard to the manufacturing process and water content of hydrophilic and hydrophobic acrylic intraocular lenses. The scratch tests were performed using a Nano Scratch Tester. A conical indenter with a tip radius of 1 µm and a cone angle of 90° was selected to scratch the samples at three different constant loads of 5, 10, and 15 mN, respectively. The scratch length was set to 100 µm at a scratch speed of 200 µm/min. Hydrophilic and hydrophobic acrylic intraocular lenses (with different water content) were tested.

Results

The results showed that for sample A (hydrophilic acrylate), the penetration depth increases steadily with increasing force from 25-30 µm (5 mN) to 28-33 µm (10 mN) and 34-37 µm (15 mN). The penetration depths during the scratches seem to be load-dependent. In sample B (hydrophobic acrylate), the same forces lead to steadily increasing penetration depths: 25-30 µm (5 mN), 40-44 µm (10 mN), and 54-57 µm (15 mN). The evaluation of the residual depth showed much lower values for all samples. In the hydrophilic, softer samples (A), the residual depth was between 1 µm and 4 µm. In the hydrophobic, more solid, samples (B), the residual depth was more pronounced with values between 5 µm and 17 µm. The plastic influence and deformation zone seemed to be wider for the hydrophobic samples than for the hydrophilic samples.

Conclusion

The laboratory experiment confirms that modern, acrylic intraocular lenses are sensitive to scratches/touch, and penetration depths during scratching depend on the load. The remaining depths after the scratches are significantly lower and show a load dependence. The deforming zone was higher in the hydrophobic acrylates than in the hydrophilic acrylates. However, the results confirm that damage can occur with hydrophobic and hydrophilic acrylic materials, depending on the force applied. Therefore, careful handling during the preparation and implantation process is crucial to prevent permanent defects.

## Introduction

Approximately >30 million cataract surgeries are performed worldwide every year [1]. After the removal of the cloudy lens using phacoemulsification, the clear, artificial intraocular lens (IOL) is implanted. This procedure has developed enormously in recent years due to various advances in technology. IOLs with new features are available for the best possible post-operative results and to further increase patient satisfaction. There are countless studies on the optical properties of lenses and also on clinical results after implantation. Adverse side effects are reported less frequently. Complications may occur if the implant is damaged during the preparation or implantation process or during positioning the IOL in the capsular bag. Defects in the material of the IOLs are of crucial importance as the lens remains in the eye for life and explantation of the damaged lens and implantation of a new lens means a great deal of effort and can entail further risks and side effects, including endophthalmitis, astigmatism, or refractive surprise, which are just a few of the side effects to be mentioned here [2-5]. For this reason, the most careful handling of IOLs during preparation and implantation is recommended. The material properties and the surface of the implant appear to be of particular importance. In recent years, preloaded injector systems have become established and have also significantly reduced the risks of damaging the lens. Nevertheless, there are still situations when scrub nurses or surgeons have to touch the intraocular lens.

The material of an IOL has to be biocompatible and durable and should combine various properties. It should be flexible so that it can be folded and the lens can be implanted into the eye through a small clear corneal incision. On the other hand, the lens should have a stable and fixed position in the capsular bag without decentration or tilt and prevent posterior capsule opacification (PCO) [6-9]. The material should also remain permanently clear without the formation of inclusions or opacities in the lens that may occur after some time (glistenings and calcifications) [10-14]. The optical properties should be as good as possible (refractive index and Abbe number) and provide optics for the best possible visual acuity and contrast sensitivity.

A variety of IOL materials are currently available, including collamer, hydrophobic acrylic, hydrophilic acrylic, polymethylmethacrylate (PMMA), PHEMA copolymer, and silicone. In a recently performed market scope analysis, hydrophobic and hydrophilic acrylates accounted for >85% of the global IOL market share for IOL materials, while PMMA accounted for 10% and silicone for 5% of the market share [15,16].

In the past, it was already shown that touching the IOL with instruments such as forceps or a spatulum can damage the surface of the IOL [17,18]. Therefore, the development of preloaded injector systems was a significant step forward in reducing the risk of damaging the lens [19]. Clinical cases have also shown that, depending on the location of scratches or Nd: YAG-associated defects, this can lead to impairment of the optical imaging quality and to straylight and increased glare [20].

It is currently assumed that the extent of the defect depends on the force and the shape of the instrument, but it is also crucially impacted by the material properties and its resistance. Acrylic lenses are produced either injection molded or with lathe-cut technique.

Depending on the water content, a distinction is made between hydrophilic and hydrophobic materials. Hydrophobicity is a measure of a particular material’s tendency to repel or separate itself from water. IOL materials are defined as hydrophobic or hydrophilic according to the angle a drop of water makes with respect to the material's surface. Hydrophilic surfaces have shallow angles while hydrophobic surfaces have larger angles [21].

Hydrophilic materials are packaged wet and typically contain 20-26% water. An IOL material is generally considered to be hydrophobic if it has a water content <5%. Traditionally, hydrophobic materials are packaged dry, but to help mitigate the effect of glistening, they have a small amount of hydrophilic character incorporated into their composition. Therefore, some of the "glistening-free" hydrophobic materials with water contents of around 5% are also supplied within saline (balanced salt solution).

In this laboratory experiment, we performed scratch tests for the mechanical assessment of acrylic intraocular lenses. We wanted to determine differences in behavior in regard to the water content.

Our study aimed to clarify whether there are differences in the sensitivity of the surfaces of modern IOL materials and whether intentionally created defects show significant differences. The derived information should help surgeons and nurses to assess whether special care is required with certain materials.

## Materials and methods

Intraocular lenses (tested samples)

We have included the following intraocular lenses in our experiment: (A) Aspira aA (HumanOptics, Germany) and (B) Envista MX 60 (Bausch & Lomb, USA). The Aspira aA is a hydrophilic, acrylic, foldable, one-piece lens with an aspheric, aberration-free optic design for improved contrast sensitivity. The optical diameter is 6.0 mm and the overall diameter is 12.5 mm with a classic C-loop haptic design. The lens has a refractive index of 1.46 and a water content of 26.0%. The IOL material is modeled on the natural lens and has been clinically proven over the course of nearly two decades. Previous studies showed that the material remains glistening-free and shows excellent uveal biocompatibility [22,23]. The Aspira aA lens is known to be very soft and flexible due to its material properties and can be implanted through small, minimal invasive clear corneal incisions (MICS). The Aspira IOL showed also good and stable positioning within the capsular bag over a long postoperative period [24]. B: The EnVista MX 60 is a hydrophobic, acrylic, foldable, one-piece lens with an aspheric, aberration-free, biconvex optic design. The optical diameter is 6.0 mm and the overall diameter is 12.5 mm with a modified C-loop haptic design with fenestrations and step vaulting. The lens has a refractive index of 1.53 (at 35°C), an Abbe number of 42, and a water content of 4.0%. Many clinical studies showed good postoperative results and a glistening-free and clear material over the years [25-27]. The EnVista MX60 lens is known to be more rigid than many competitors due to the material properties and relatively low water content and therefore very stable within the capsular bag. All tested samples had the same IOL power (21.0 D). It should be noted that the most obvious differences in the samples are the water content and the manufacturing process.

Scratch tests

**Figure 1:**
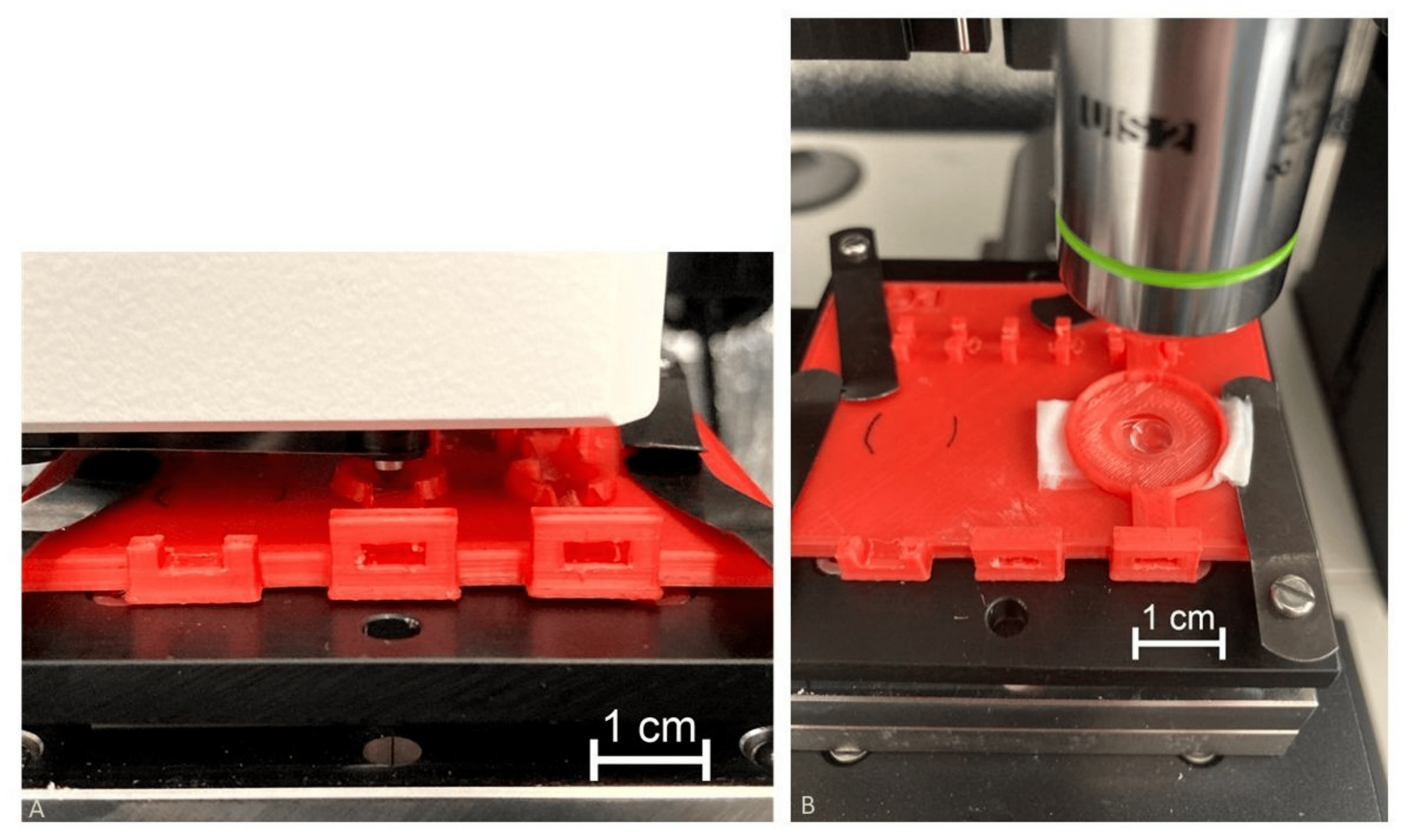
Test setup of the indenter. The intraocular lens (IOL) samples were placed in the middle of the customized sample holder in the nano scratch tester (A). Afterward, the scratches were analyzed using light microscopy (B). Image credits: Andreas F. Borkenstein and Eva-Maria Borkenstein

A customized sample holder was designed to perform scratch tests on the samples. This sample holder clamps the stabilizing arms of the IOLs and simultaneously provides enough space for the indenter and the objective of the positioning light microscope. The IOL was placed in the middle of the sample holder, and to prevent any tilting, the stabilizing arms were supported by wet wipes (Figure 1).

The scratch tests were performed using an NST³ Nano Scratch Tester (Anton Paar, Austria). A conical indenter with a tip radius of 1 µm and a cone angle of 90° was selected to scratch the samples at three different constant loads of 5, 10, and 15 mN, respectively. The scratch length was set to 100 µm at a scratch speed of 200 µm/min. To minimize the influence of the deformation, scratches with the same load were offset by 300 µm in horizontal direction. The scratches with loads of 10 and 15 mN were stacked over the original scratches with an offset of 300 and 600 µm in the vertical direction. The six scratches per lens were performed in one batch. Immediately afterward, the lens was examined in a laser scanning confocal microscope (VK-X1000, Keyence, Japan). In order to better compare the individual scratches, the curvature of the lens was removed using software. In principle, the Nano Scratch Tester works as follows: Before each test, the surface is scanned within a pre-scan. Here, the indenter is loaded with a minimal force used to determine surface roughness or tilt of the sample, which can then be compensated during the actual test. During the scan a scratch is performed with a constant load and the penetration depth is measured. Finally, during the post-scan, the indenter traces again the scratch from the starting point to the end and records the residual depth [28,29]. After the sample has been brought into focus under the microscope, the sample is moved under the indenter. Six scratches were measured in the following grid: scratches of the same load horizontally (distance 300 µm), the next higher load was shifted by 300 µm in the y-direction with the same x-coordinate.

Test procedure (Prescan, Scan, and Postcan)

In the Prescan, the sample surface is scanned to obtain a profile. The Scan is the actual measurement during which the penetration depth (depending on the load) is measured to assess the penetration depth. At the end, the Postscan is performed: after the measurement, the scratch is scanned again with the indenter (in the same direction) to determine the remaining depth (residual depth). The following measurement parameters were used: constant load (FN): 5 mN, 10 mN, and 15 mN; scratch length: 100 µm; scanning speed: 200 µm/min; indenter tip: conical indenter with a tip radius of 1 µm. All scratches were carried out in a patch to prevent the lens from drying out.

## Results

The scratch tests with three different loads (5, 10, and 15 mN) were successfully performed on two different acrylic intraocular lenses (samples A and B).

**Figure 2:**
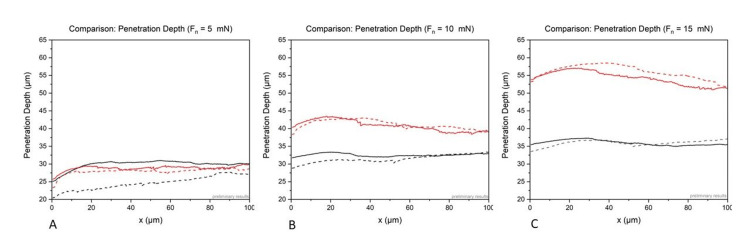
Comparison of the results of hydrophilic, acrylic samples (black) and hydrophobic, acrylic samples (red) in regard to penetration depth and different loads with Fn = 5 mN (A), Fn = 10 mN (B), and Fn = 15 mN (C).

The comparison of the penetration depth showed that for sample A (hydrophilic acrylate), the penetration increases steadily with increasing force from 25 µm to 30 µm (5 mN) to 28-33 µm (10 mN) and 34-37 µm (15 mN). In sample B (hydrophobic acrylate), the same forces lead to steadily increasing penetration depths: 25-30 µm (5 mN), 40-44 µm (10 mN), and 54-57 µm (15 mN). Figure 2 shows a comparison of the hydrophilic and hydrophobic samples.

**Figure 3:**
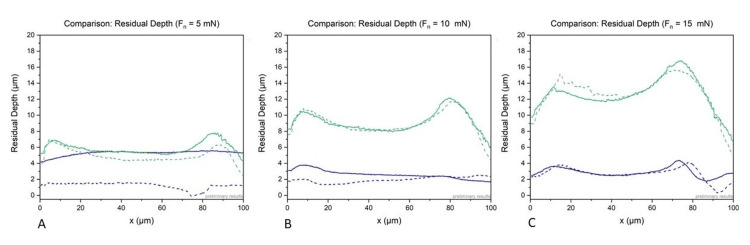
Comparison of the results of the hydrophilic, acrylic samples (blue) and hydrophobic, acrylic samples (green) in regard to the residual depth and different loads with Fn = 5 mN (A), Fn = 10 mN (B), and Fn = 15 mN (C).

The evaluation of the residual depth showed much lower values for all samples. In the hydrophilic, softer sample (A), the residual depth was between 1 µm and 4 µm. In the hydrophobic, more solid, sample (B), the residual depth was more pronounced with values between 5 µm and 17 µm. Figure 3 shows a comparison of the hydrophilic and hydrophobic samples.

**Figure 4:**
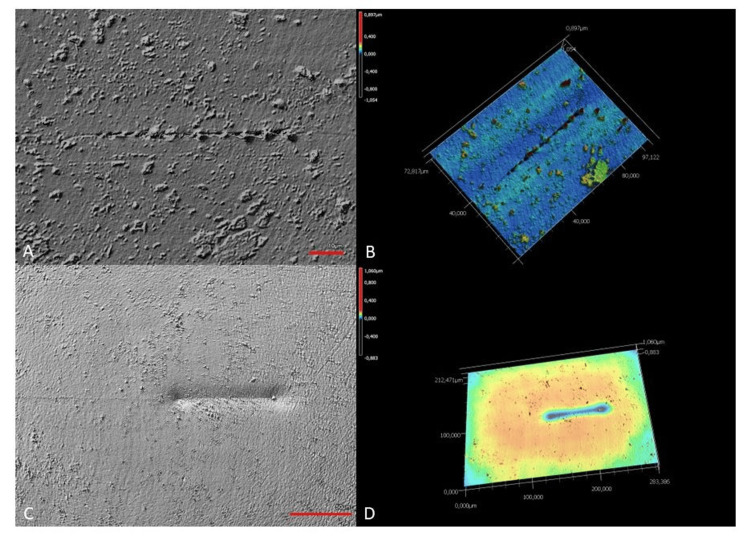
Analyzing the scratches using differential interference contrast microscopy (left) and 3D reconstruction (right). An example of a hydrophilic acrylic sample (A, B) and a hydrophobic acrylic sample (C, D). Image credits: Andreas F. Borkenstein and Eva-Maria Borkenstein

This is a clear sign that the penetration depths during the scratches are load-dependent. The remaining depths after the scratches are considerably lower and showed a load dependency, especially with sample B. The optical evaluation in differential interference contrast showed the increasing depth of the scratches with the load (Figure 4).

The plastic influence and deformation zone was wider for sample B than for sample A.

## Discussion

Scratch testing is the most widely used test for evaluating the adhesion strength of coating-substrate systems. During the test, a spherical diamond indenter with a specific radius is used to exert a continuously increasing force on the coating surface while the sample is moved at a constant speed. The scratching of the surface leads to increasing elastic and plastic deformation until the coating largely flakes off the substrate at a certain critical load (Lc). The hardness of the substrate material has a significant effect on the scratch resistance (cracking) of thin coatings during testing.

Superficial injuries to modern acrylic intraocular lenses can occur both during preparation and implantation. The development of fully preloaded injectors in recent years has reduced the risk of such defects. However, after the folding and injection process, some superficial scratches and tears of the IOL optic were still detected in some models. Studies showed that the unintentional extensive pressure effect of the forceps during the holding process of the IOL or the direct trauma to the lens optic by the plunger of injector systems may cause optic cracks or fractures [30]. These defects can lead to glare, image degradation, and visual disturbances. These results and the effect on the optical properties of the IOL are consistent with other defects that can occur not only mechanically by contact but also by laser energy during YAG capsulotomy and a misfocused laser aiming beam [31]. The effect of such defects appears to be even greater in presbyopia-correcting IOLs. In small-aperture (pinhole) IOLs, the incorrect use of the laser beam can even lead to massive destructive effects [32,33]. Further studies should therefore follow to find out the differences between the materials and increase awareness among surgeons. It can already be summarized that the material of modern, acrylic IOLs is sensitive and inappropriate contact (mechanical or laser) should be avoided. Manufacturing companies should also consider these factors and think about possible safety features (e.g., less sensitive surface and coating).

## Conclusions

The scratch tests carried out with varying loads (5, 10, and 15 mN) on samples A and B have shown a clear load dependence of the penetration depths during the scratch process. The remaining depths after the scratches are significantly smaller and, particularly with lens B, depend on the applied load. The residual depth in the hydrophilic samples was between 1 µm and 4 µm. In the hydrophobic, more solid, samples, the residual depth was more pronounced with values between >5 µm and 17 µm. The optical evaluation using differential interference contrast microscopy showed an increasing depth of the scratches with increasing load. In addition, the plastic influence and deformation zone in lens B appeared wider than in lens A, which indicates a higher susceptibility of lens B to mechanical stress. These results highlight the relevance of material properties in determining the performance of optical lenses under various mechanical loads. There are IOL materials that may react even more sensitively to scratches, but with all modern acrylic lenses, any unnecessary contact with the lens optics during the preparation and implantation process should be avoided.

The laboratory experiment has shown that acrylic materials with different water content and manufacturing processes are more or less sensitive to external force. However, defects can be created on all materials. It is not possible to give a ranking of the tested lenses from good to bad based on the results. All tested samples have confirmed their quality through numerous clinical studies since their market launch. The key message of this lab experiment is that the knowledge about the vulnerability of modern, foldable acrylic lenses seems to be important to achieve the best postoperative results in clinical routine and avoid any permanent defects.
